# A Smartphone App to Improve Intuitive Eating and Diet Quality: Design and Usability Study

**DOI:** 10.2196/81439

**Published:** 2026-07-17

**Authors:** Mandy Korpusik, Delaram YazdanSepas, Hawley C Almstedt

**Affiliations:** 1 Department of Computer Science Seaver College of Science and Engineering Loyola Marymount University Los Angeles, CA United States; 2 Department of Health and Human Sciences Seaver College of Science and Engineering Loyola Marymount University Los Angeles, CA United States

**Keywords:** disordered eating, nutrition, college age, intuitive eating, mobile app, natural language processing, recommendations, recipes, physical activity

## Abstract

**Background:**

Web-based and mobile phone–based apps have become widely available for dietary self-monitoring; however, their use may increase the risk of disordered eating. College students frequently demonstrate poor nutrient intake despite consumption of sufficient calories. One way to improve diet quality may be via the use of a smartphone app that encourages intuitive eating.

**Objective:**

The purpose of this study was to improve diet quality among college students through the use of a novel smartphone app that promotes intuitive eating rather than calorie counting and weight loss.

**Methods:**

The In2Eat iOS mobile app was developed in SwiftUI and stored user data in a Firebase database. A total of 45 college students completed assessments of intuitive eating, diet quality, and disordered eating before and after 4 weeks of using the In2Eat app. Users evaluated the usability of the app with the System Usability Scale (SUS). Engagement with the app was recorded as the total number of days a meal was logged, the total number of meals logged, and the average number of meals logged per day.

**Results:**

After our 4-week intervention, dietary qualities that protect against chronic disease increased by 28%, fruit consumption increased by 63%, and skin antioxidant levels increased by 6.1% (Hedges *g*=0.16; mean difference 0.33, 95% bias corrected and accelerated [BCa] CI 0.04-0.61; *P*=.03). Global intuitive eating did not change during the user study; however, the unconditional permission to eat subscale increased (Hedges *g*=−0.28; mean difference 0.28, 95% BCa CI 0.07-0.49; *P*=.01, adjusted *P*=.07). Overall, disordered eating also did not change with app use, although dietary restraint decreased (Hedges *g*=−0.23; mean difference 0.30, 95% BCa CI −0.61 to −0.04; *P*=.04, adjusted *P*=.22). The average SUS score for the In2Eat app was 67.2 (SD 15.5). The number of days a meal was logged was positively correlated with SUS scores (*r*=0.28; *P*=.06), and the total number of meals logged had a monotonic association with app usability (ρ=0.31; *P*=.04). When divided according to the low (mean 10.2, SD 5.3), medium (mean 26.3, SD 2.8), and high (mean 33.6, SD 3.8) number of days logging meals, participants with higher days of logging reported the app as more usable (*H*=6.75; *P*=.03). A regression analysis showed that 8% of the variance in system usability (*R*^2^*=*0.080; *P=*.31) was explained by app use; however, none of the individual predictors contributed substantially to the variance.

**Conclusions:**

An intuitive eating smartphone app can improve diet quality without increasing disordered eating. Results suggest that participants who logged more meals tended to rate the app as more usable. Further research is needed with a greater sample size after incorporating features to improve the app’s usability.

## Introduction

Web-based and mobile phone–based apps have become widely available for dietary self-monitoring, with some incorporating recent technological advances, such as speech recognition for logging meals with voice and computer vision for food image understanding to reduce user burden [1-3]. In our prior work, we developed an iOS app called COCO Nutritionist that used natural spoken language understanding and machine learning to automate mapping of self-reported meal logs to the US Department of Agriculture (USDA) food codes and portion sizes, and we demonstrated in a pilot study that there was no significant difference in 3-day reported energy intake between our method and gold-standard 24-hour recalls [4].

Previous research shows that lifestyle habits developed as a college-aged young adult have a lasting influence on physical health, mental health, and general well-being [5,6]. Despite this, there is ample literature demonstrating inadequate nutrient intake among college students [7,8]. Malnutrition exists when there is a lack of calorie intake (ie, undernutrition) and when dietary intake is excessive. Of particular concern is a high intake of calories lacking in nutritional quality, increasing risk for chronic disease. Vitamin and mineral deficiencies can occur even when the diet is abundant in calories; hence, the need to assess diet quality, not just calorie intake [9]. Diet quality assesses the extent to which a diet has sufficient health-promoting foods to avoid vitamin and mineral deficiencies and protect against chronic disease while avoiding overconsumption of dietary factors that increase risk for chronic disease.

One way to improve diet quality may be via the use of a smartphone app that encourages healthy eating. However, research suggests that use of smartphone apps that track calorie intake is related to disordered eating symptoms [10,11]. Repetitive monitoring of calorie intake may lead to obsessive behaviors, anxious feelings, and unhealthy dietary restrictions. Intuitive eating is a conceptual eating behavior that emphasizes the use of internal cues for hunger and satiety to guide food choices [12]. Preoccupation with food, restriction, and rigidity are commonly observed with disordered eating. However, an intuitive approach to eating encourages achieving satisfaction without overeating by using interoceptive awareness while allowing unconditional permission to eat. The 10 principles of intuitive eating include rejecting diet culture, honoring hunger, making peace with food, discovering satisfaction, feeling satiety, challenging diet “rules,” coping with emotions, respecting bodies, joyful movement, and honoring health [12]. Research has found that the use of intuitive eating principles may counteract disordered eating behaviors [13-16].

The purpose of this study was to improve diet quality among college students via a smartphone app designed to encourage intuitive eating and recommend personalized healthy recipes. We hypothesized that consistent use of our smartphone app over 4 weeks would improve diet quality without increasing disordered eating.

## Methods

This paper describes the design, development, and evaluation of, to our knowledge, the first mobile app for automated self-report of dietary intake via natural spoken language that promotes intuitive eating principles.

### App Development

The In2Eat iOS mobile app was developed by a team of undergraduate computer scientists at Loyola Marymount University. In the preliminary design phase, 2 other apps were found to promote intuitive eating—MyTummy and MEAL. However, neither of these apps supported the kind of self-reported dietary intake and detailed nutrient information that was common among popular diet-tracking apps such as MyFitnessPal, Noom, and Weight Watchers. In2Eat is unique in that it both supports low-burden diet logging and promotes intuitive eating. Diet tracking is required for improving diet quality, which is our goal and is associated with health and disease prevention. However, our intuitive eating approach aims to avoid fostering disordered eating that can occur with apps that emphasize weight loss and calorie counting [10,11].

In2Eat’s natural language processing technology for easily logging meals with voice or text was originally developed for the COCO Nutritionist research prototype [4]. COCO used machine learning, specifically convolutional neural networks (CNNs), to accomplish 2 tasks: parsing a user’s natural language meal description into foods and quantities and mapping the user’s food descriptions to a matching entity in the validated USDA food database using learned embeddings. The CNN automatically learned semantic vector representations, or embeddings, of the user’s unstructured natural language meal description through a binary classification task where the model learned to detect which pairs of USDA foods and meal descriptions were a match. With In2Eat, the 2-step approach of parsing a user’s meal description, followed by database mapping with embeddings, is still the same, but the implementation now uses large language models (LLMs) for parsing meal descriptions and embeddings from LLMs instead of CNNs.

We built our In2Eat app in SwiftUI and stored user data in a Firebase database. A key component of our intuitive eating design is that the app does not display caloric information and instead focuses on diet quality by reporting consumed macro and micronutrients. In addition, there is a daily push notification with an intuitive eating quote of the day, which is drawn from intuitive eating content that we curated. This intuitive eating quote of the day is also displayed on the main dashboard screen upon opening the app. In2Eat supports food logging via written or spoken natural language, along with a hunger satiety check-in at each meal. Several principles of intuitive eating can be fostered by increasing awareness of feelings of hunger and satiety before or after eating occasions. Thus, experts coach intuitive eating by using a scale of 0 to 10 to represent primal or painful hunger (as 0) and painfully full or stuffed (as 10) [12]. The macronutrients are shown in a pie chart on the dashboard, and the micronutrients are shown as green, yellow, or red progress bars toward reaching the recommended amount based on the user’s sex and weight. The recommended targets were based on the Dietary Reference Intakes established by the Institute of Medicine [17]. In2Eat’s recipe book recommends healthy recipes, defined as at least 5 g of fiber and no more than 5 g of saturated fat per serving that align with the user’s self-reported dietary restrictions, when applicable. Users can save favorite recipes and search for specific recipes as well as filter by cooking and preparation time, cuisine, price, and allergens. Finally, there is a screen for logging the number of minutes of physical activity, without having to label the type of exercise, which aligns with the “joyful movement” principle of intuitive eating.

### Recruitment

College-aged adults were recruited via flyers and classroom announcements at a primarily undergraduate institution in an urban setting. Advertising flyers were specifically placed on the walls in the on-campus food pantry, which is available for access 24 hours a day, 7 days per week. Inclusion criteria included being aged 18 years or older and having access to an iOS smart device.

A total of 47 volunteers enrolled in the study; however, 2 (4.3%) were lost to follow-up because they did not use the app and were unresponsive when attempting to schedule follow-up testing. Therefore, 45 (95.7%) participants were included in this analysis. At the time of enrollment, participants completed several online questionnaires and came into the laboratory for measurements of height, weight, and skin antioxidant levels. During the first laboratory visit, participants downloaded the In2Eat app onto their smart device and were given brief instructions on how to use the app. Volunteers were instructed to use the app for the next 4 weeks. After 4 weeks of app use, participants repeated the online questionnaires, as well as height, weight, and skin antioxidant level assessments. Incentives of US $50 grocery store gift cards were provided to participants who completed the baseline and follow-up testing sessions.

### Measures

#### System Usability

The System Usability Scale (SUS) was used at follow-up to assess the app’s usability on a scale from 0 to 100. The average SUS score is 68, with 68 through 80.3 considered good and greater than 80.3 considered excellent [18,19]. The instrument consists of 10 questions (eg, “I thought the app was easy to use”), each with 5 options ranging from strongly agree to strongly disagree.

#### App Use

To investigate the relationship between app engagement and perceived usability, we computed 3 use metrics: LogCount (total number of days a participant logged at least 1 meal), TotalMealsLogged (total number of meals logged throughout the study), and AvgMealsPerDay (mean number of meals logged per day, calculated as TotalMealsLogged divided by LogCount). These metrics capture both breadth and depth of engagement with the app.

#### Intuitive Eating

Intuitive eating was assessed using the Intuitive Eating Scale version 3 (IES-3) established for reliability and validity [20]. Possible results for the IES-3 range from 1 to 5 with higher scores indicating greater intuitive eating. Scoring the survey provides an overall global score for intuitive eating as well as scores on 4 subscales: unconditional permission to eat, reliance on hunger and satiety cues, eating for physical reasons, and body food choice congruence.

#### Disordered Eating and Mental Health

The Eating Disorder Examination Questionnaire (EDE-Q) was used to assess disordered eating patterns. This validated and internally consistent questionnaire provides a global disordered eating score and 4 subscales for dietary restraint, eating concern, body shape concern, and weight concern [21,22]. Dietary restraint reflects a tendency to consciously restrict or limit dietary intake. Eating, body shape, and weight concern are subscale scores that reflect an unhealthy preoccupation or worry with these features of the diet or body. The EDE-Q results range from 0 to 6, with higher scores indicating greater disordered eating. Volunteers were also asked to complete the Kessler Psychological Distress Scale (K10) with 10 prompts, scored from 1 to 5 and summed. Results from the K10 range from 10 to 50 with higher scores indicating severe mental disorder [23,24].

#### Diet Quality

Volunteers submitted the Diet Quality Questionnaire (DQQ), which is validated for use by adults in more than 100 countries [25]. This categorical survey uses food group indicators to calculate how well the diet achieves a global dietary recommendation (possible scores range from 0 to 18) and the degree to which dietary intake on the previous day protects against noncommunicable diseases (NCD-Protect, possible scores range from 0 to 9) and increases risk for noncommunicable diseases (NCD-Risk, possible scores range from 0 to 9). The DQQ also quantifies the proportion of the study group who ate at least 1 serving from all 5 of the food groups recommended in global food-based dietary guidelines (vegetable, fruit, pulse, nuts and seeds, animal-source food, and starchy staple). Diets of high quality also exhibit diversity; thus, the dietary diversity score ranges from 0 to 10 to reflect the average score for the study group, with a higher score indicating the presence of more food groups. Finally, the DQQ quantifies the proportion of the study group who consumed at least one fruit and one vegetable on the preceding day. Data from our investigation were compared against results collected from adults in the Global Diet Quality Project between 2021 and 2023 [26].

As an objective measurement of diet quality, antioxidant levels in the skin were evaluated using a handheld spectrophotometer (Konica Minolta CM-700d). More specifically, fruits and vegetables contain carotenoids that act as antioxidants to reduce inflammation and cell damage [27]. Carotenoids, and specifically β-carotene, present as a yellow color and typically absorb light in the 450-nm frequency [28]. A spectrophotometer can be used to measure light reflection at specific wavelengths as an indicator of carotenoids, and therefore fruit and vegetable consumption. Using a previously documented noninvasive approach, we recorded the tristimulus b* value at the 450-nm frequency to assess carotenoid levels in the skin [29]. After daily calibration, the thenar eminence of the palm of the hand for each participant was scanned twice with the spectrophotometer. An average of the 4 scans was calculated and used for statistical analysis. As carotenoids are found in fruits and vegetables, measuring skin carotenoid levels is an accurate and less invasive measurement of dietary intake compared with self-reported data or blood tests.

#### Food Security

Much research has suggested that food security is directly related to diet quality [30]. Thus, we used the USDA Food Security Survey to assess whether our participants had limited or uncertain availability of nutritionally adequate foods [31].

### Statistical Analysis

We examined associations between these metrics and SUS scores using correlation and regression analysis. Correlation (Pearson and Spearman) and ordinary least squares regression were conducted using Python (version 3.11; Python Software Foundation) with the Pandas, SciPy, Seaborn, and Statsmodels libraries. To assess the combined predictive value of use behaviors, we included all 3 use metrics as predictors in the regression model. Paired sample, 2-tailed *t* tests were performed using SPSS software (version 29; IBM Corp) to evaluate differences between variables at baseline and after 4 weeks of app use. Bootstrapping was performed with 5000 samples and bias corrected and accelerated (BCa) CI were calculated. Cohen *d* and Hedges correction were calculated as measures of the effect size. The Holm-Bonferroni adjustment was made to correct for multiple comparisons. Assumption testing was performed for outcomes yielding statistically significant results using the Shapiro-Wilk test and Q-Q plots. Where the normality assumption did not hold, a Wilcoxon signed-rank test was conducted as a sensitivity analysis. Missing data were excluded.

### Ethical Considerations

Participants were fully informed of the study protocol and provided written informed consent before enrolling. This study was approved by the Loyola Marymount University Institutional Review Board (protocol number 2024FA16-R) according to the Declaration of Helsinki. Participants were assigned an identification number to protect privacy and ensure confidentiality.

## Results

### Overview

Demographic data for the 45 participants who completed the study are displayed in Table 1. This college-aged group was made up primarily of female participants (n=33, 73.3%) from diverse ethnic and racial groups, with approximately half living on campus (n=22, 48.9%) and using the campus-offered meal plan (n=24, 53.3%). Of the 45 participants in the study, 5 (11.1%) reported low (n=4, 8.8%) or very low (n=1, 2.2%) food security at baseline and follow-up. Food security measures did not correlate with intuitive eating, disordered eating, or diet quality in this population.

**Table 1:** Demographic characteristics of 45 college-aged volunteers who used the In2Eat app for 4 weeks.

			Value
Age (y), mean (SD)			20.5 (1.2)
Height (cm), mean (SD)			165.2 (8.3)
Weight (kg), mean (SD)			64.6 (13.5)
**Sex, n (%)**			
	Female	33 (73.3)	
	Male	12 (26.7)	
**Ethnicity, n (%)**			
	Hispanic	13 (28.9)	
	Non-Hispanic	32 (71.1)	
**Race, n (%)**			
	Asian	15 (33.3)	
	Black or African American	10 (22.2)	
	Mixed (Asian and White)	5 (11.1)	
	White	10 (22.2)	
	Declined to state	5 (11.1)	
**Year in college, n (%)**			
	First year	7 (15.6)	
	Sophomore	18 (40)	
	Junior	9 (20)	
	Senior	11 (24.4)	
**Living status, n (%)**			
	On campus	22 (48.9)	
	Off campus	23 (51.1)	
**Meal plan^a^** **, n (%)**			
	Yes	24 (53.3)	
	No	21 (46.7)	

^a^Refers to whether students used the campus-offered meal plan.

### System Usability

**Figure 1:**
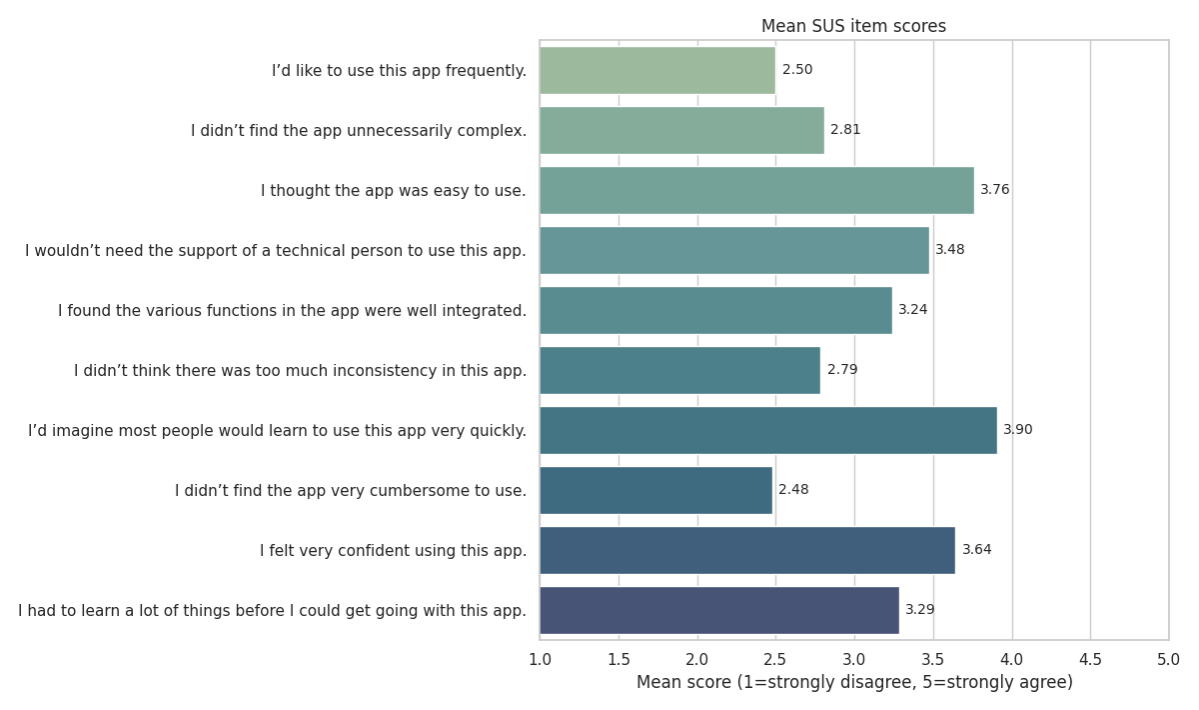
System Usability Scale (SUS) scores for each of the 10 survey questions, ranging from the lowest score of 1 (“Strongly disagree”) to the highest score of 5 (“Strongly agree”).

The average overall system usability score for the In2Eat app at the end of the 4-week pilot study was 67.2 (SD 15.5), which is similar to the commonly cited SUS benchmark score of 68 [18,19]. As shown in Figure 1, the questions with the highest scores were those concerning ease of use, specifically that users thought people would quickly learn to use the app, the app was very easy to use, and they felt confident using the app. By contrast, the questions with the lowest scores were those related to not finding the app cumbersome to use, wanting to use the app frequently, and not finding too much inconsistency or complexity in the app.

### App Use

**Figure 2:**
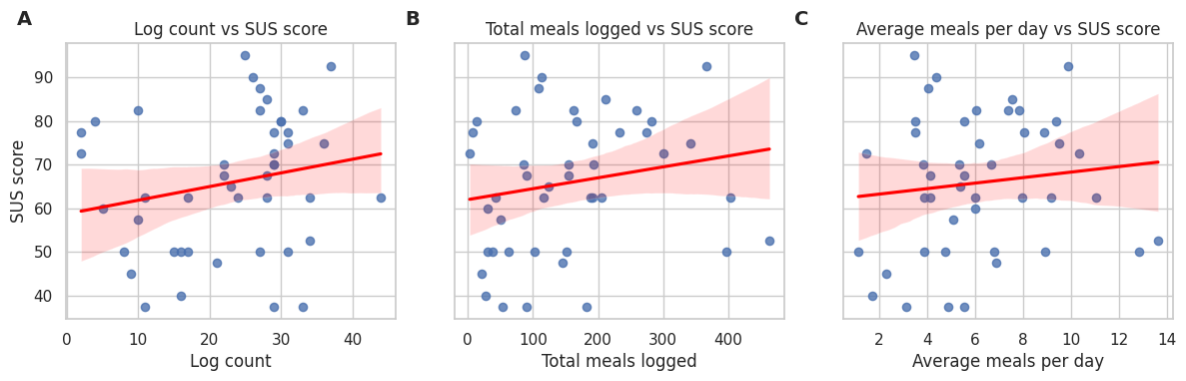
Relationship between app use metrics and perceived system usability. Each panel shows a scatterplot with a linear regression line, comparing (A) the total number of days users logged meals (LogCount), (B) the total number of meals logged, and (C) the average number of meals per day with the System Usability Scale (SUS) scores. Across all 3 use metrics, greater engagement with the app tended to be associated with higher perceived usability.

**Figure 3:**
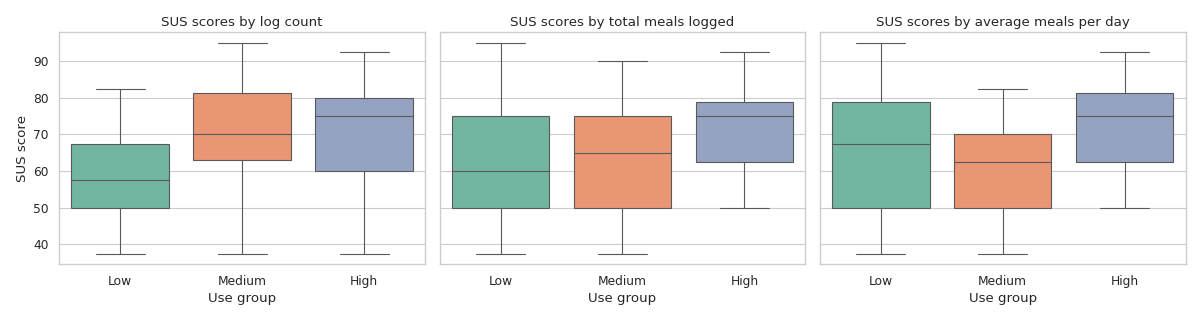
Distribution of System Usability Scale (SUS) scores grouped by use level (low, medium, and high) for each app engagement metric. Participants with higher app use more frequently reported higher usability scores, particularly those with higher LogCount values.

Figures 2 and 3 visualize the associations between system usability scores and app use using both scatterplots and group-wise boxplots. Pearson and Spearman correlations showed modest and mostly nonsignificant associations between use metrics and SUS scores. LogCount was positively correlated with SUS scores (Pearson *r=*0.28; *P*=.06), though this did not reach conventional significance. Notably, TotalMealsLogged had a statistically significant monotonic (but not linear) association with SUS scores (Spearman ρ=0.31; *P*=.04), suggesting that participants who logged more meals tended to rate the app as more usable.

Table 2 presents descriptive statistics for app use across the full study sample as well as within low, medium, and high use groups (tertiles based on LogCount). As expected, participants in the high use group demonstrated consistently greater engagement across all metrics compared with those in the medium and low use groups. Specifically, high use users logged meals more frequently, entered a greater total number of meals, and maintained a higher average number of meals logged per day. The medium use group showed intermediate values across all engagement metrics. These results confirm a clear separation between groups, capturing both the breadth and intensity of app engagement across the cohort.

Tertile grouping was used to provide an interpretable comparison of usability across distinct levels of engagement (low, medium, and high) and to examine potential nonlinear patterns in the relationship between app use and perceived usability that may not be captured by correlation analyses alone. In addition to group-based comparisons, we report both Pearson and Spearman correlations between use metrics and SUS scores. These analyses showed modest associations, with a statistically significant monotonic relationship observed between TotalMealsLogged and SUS scores (Spearman ρ=0.31; *P*=.04), indicating that greater meal logging was associated with higher perceived usability.

We further explored differences in usability scores across low, medium, and high use groups (tertiles based on LogCount). A Kruskal-Wallis test revealed a significant difference in SUS scores across LogCount groups (*H*=6.75; *P*=.03), indicating that participants with higher day-level logging frequency perceived the app as more usable. No substantial group differences were observed for TotalMealsLogged or AvgMealsPerDay.

Finally, we performed an ordinary least squares regression, including all 3 use metrics as predictors of SUS scores. The overall model explained only 8% of the variance (*R*^2^=0.080; *P*=.31), and none of the individual predictors were statistically significant. While LogCount had the largest coefficient (β*=*.55), its contribution was not statistically robust (*P*=.31). These results suggest a trend toward more consistent engagement being associated with higher usability ratings, but the effect sizes are modest and should be interpreted cautiously given the limited sample size.

**Table 2:** App use metrics among the 45 participants overall and by group.

Use metrics	Overall, mean (SD)	Low, mean (SD)	Medium, mean (SD)	High, mean (SD)
LogCount	23.11 (10.34)	10.2 (5.28)	26.28 (2.76)	33.62 (3.8)
TotalMealsLogged	157.09 (115.43)	57.8 (52.95)	144.17 (64.41)	289.54 (96.13)
AvgMealsPerDay	6.19 (2.85)	5.02 (2.66)	5.45 (2.2)	8.57 (2.59)

### Intuitive Eating

The global score for intuitive eating did not change after 4 weeks of using the In2Eat app (Table 3); however, the unconditional permission to eat subscale of the IES-3 did increase significantly (*P*=.01), though with a small effect size (Hedges *g*=−0.28) and not after Holm-Bonferroni adjustment to control for type I error across related outcomes with multiple comparisons (adjusted *P*=.07). Normality assumptions were met.

**Table 3:** Intuitive eating, disordered eating, and psychological distress at baseline and after 4 weeks (n=42).

Outcome			Baseline, mean (SD)		Mean difference^a^ (95% BCa^b^ CI)		*P* value		Adjusted *P* value^c^		Hedges *g*
**Intuitive eating**											
	Global score	3.78 (0.53)		0.02 (−0.08 to 0.12)		.68		.68		0.04	
	Unconditional permission to eat	3.60 (1.03)		0.28 (0.07 to 0.49)		.01^d^		.07		0.28	
	Eating for physical reasons	3.83 (0.75)		−0.06 (−0.23 to 0.13)		.55		>.99		−0.08	
	Reliance on hunger satiety cues	3.79 (0.74)		−0.15 (−0.34 to 0.02)		.12		.48		−0.19	
	Body food choice congruence	3.89 (0.60)		0.05 (−0.08 to 0.17)		.49		>.99		−0.08	
**Disordered eating**											
	Global score	1.24 (1.02)		−0.10 (−0.31 to 0.09)		.32		>.99		−0.10	
	Dietary restraint	0.96 (1.38)		−0.30 (−0.61 to −0.04)		.04^d^		.22		−0.23	
	Eating concern	0.66 (0.65)		−0.05 (−0.21 to 0.12)		.55		>.99		−0.07	
	Body shape concern	1.77 (1.41)		−0.05 (−0.33 to 0.21)		.74		>.99		−0.03	
	Weight concern	1.56 (1.34)		−0.03 (−0.33 to 0.25)		.84		.84		−0.02	
Psychological distress			17.0 (5.23)		−0.69 (–2.45 to 1.02)		.41		—^e^		−0.13

^a^Mean difference was calculated as week 4−baseline.

^b^BCa: bias corrected and accelerated.

^c^Holm-Bonferroni adjusted *P* value.

^d^Statistical significance at *P*<.05.

^e^Not applicable.

### Disordered Eating and Mental Health

Internal consistency at baseline was excellent for the EDE-Q scale (α=0.95) and the K10 (α=0.89). Baseline and follow-up assessments of disordered eating via the EDE-Q revealed no major changes during app use, except for dietary restraint which decreased significantly (*P*=.04), though with a small effect size (Hedges *g*=−0.23) and not after Holm-Bonferroni adjustment to control for familywise error (adjusted *P*=.22). For dietary restraint, the normality assumption did not hold due to an S curve in the Q-Q plot and the Shapiro-Wilk test result (*P*<.001); however, the Wilcoxon signed-rank sensitivity analysis (*P*=.03) confirmed the finding, indicating that the result was robust to nonnormality.

### Diet Quality

Diet quality among app users also appeared to improve during the study (Table 4). Dietary features that are known to protect against noncommunicable diseases (NCDs) increased by 28%, while there was a negligible decrease (4%) in dietary features that increase the risk for NCDs. Of particular note is the 63% increase in fruit consumption during the app intervention. Furthermore, paired *t* tests (N=47) revealed a significant increase in skin antioxidant levels (*P*=.03) with a small effect size (Hedges *g*=0.16).

We additionally examined whether outcomes differed between students living on campus and those living off campus. No significant differences were observed between groups across all primary outcomes, including diet quality (global dietary recommendation score, NCD-Protect, and NCD-Risk), intuitive eating (IES-3), disordered eating (EDE-Q), and skin antioxidant levels (all *P*>.05). Similarly, SUS scores did not differ between groups (*P*=.36). These findings suggest that residential status did not meaningfully influence study outcomes.

**Table 4:** Diet quality assessed before and after 4 weeks of using the In2Eat app.

	Baseline	4 weeks	Change (%)	United States average^a^
Global dietary recommendations	9.13	9.98	9.3	9.5
NCD^b^-protect^c^	2.63	3.37	28.1	3.9
NCD-risk^d^	2.50	2.39	−4.3	3.5
All-5^e^	0.21	0.28	34.6	0.44
Dietary diversity^f^	5.19	5.43	4.7	6.4
At least 1 fruit^g^	0.44	0.72	63.0	0.77
At least 1 vegetable^h^	0.71	0.70	−2.0	0.85
Skin antioxidant levels	15.38	15.71	6.1	—^i^

^a^Average scores for adults in the United States [26].

^b^NCD: noncommunicable disease.

^c^Degree to which diet is protective against noncommunicable diseases.

^d^Degree to which diet increases risk for noncommunicable diseases.

^e^Proportion of participants who ate all 5 food groups on the previous day.

^f^Number of food groups consumed on the previous day.

^g^Proportion of participants eating at least one fruit on the previous day.

^h^Proportion of participants eating at least one vegetable on the previous day.

^i^Not applicable.

### Qualitative Feedback

**Figure 4:**
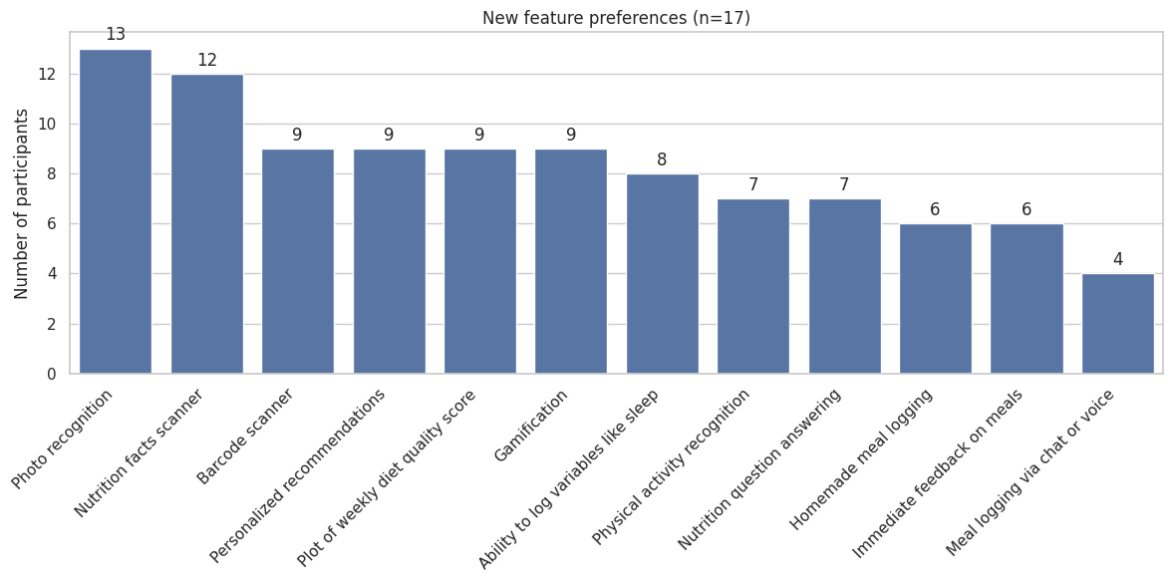
Frequency of interest in hypothetical new features from 17 app users.

We collected qualitative feedback from 17 study participants via a Qualtrics survey to inform future strategies for improving the app and to investigate why participants did not frequently use the app despite the monetary incentive. The survey asked 2 free-response questions, one about why they may not have used the app often, and the second about how we could improve the In2Eat app. The final question asked them to select which of 12 hypothetical new features would make them more likely to use the app more often. As shown in Figure 4, by far the most popular new feature was computer vision—13 (76.5%) out of 17 respondents selected the ability to log photos of their meals, and 12 (70.6%) selected the ability to take a photo of a nutrition facts label.

Patterns emerged from the free-response questions as well. Four (23.5%) respondents each mentioned forgetting to log meals and that reminders would be helpful, that they would like incentives or rewards for logging meals, and that the data entry was too time-consuming and/or annoying. Three (17.6%) respondents each mentioned wanting computer vision; worries about their own logging precision, especially portion size, as well as the accuracy of the app itself; and the app missing foods, including non-American cuisines. Other feedback included speeding up meal logging with “quick add” of common foods or meals; making the interface more interactive, visual, and informative; adding a social tab to see friends; helping them to track and achieve their nutrition goals; and adding a barcode scanner. This qualitative data analysis strongly supported our current work on incorporating computer vision as well as gamification features (ie, incentives, goals, and friends).

## Discussion

### Principal Findings

The overarching goal of this study was to investigate whether the use of a mobile phone app that promotes intuitive eating principles (through hunger satiety checks at each meal and push notifications with daily intuitive eating quotes of the day) would improve diet quality, without increasing disordered eating symptoms as often occurs when using other diet-tracking apps [10,11]. Our results from a 4-week user study with 45 college students demonstrated that diet quality improved, as measured by a significant increase in skin antioxidant levels [27], a 63% increase in fruit consumption, and a 28% increase in dietary features known to protect against NCDs. Compared with data reported for American adults, a smaller proportion of our college-aged participants consumed all 5 food groups, 1 fruit, 1 vegetable, and showed a lower dietary diversity score. On a positive note, the global dietary recommendation score for our population was above the national average for US adults, while the NCD-Protect score became closer to the national average after 4 weeks of app use. Notably, the NCD-Risk score among our participants was lower than national norms, indicating a diet with fewer contributing factors to disease. The unconditional permission to eat subscale of the intuitive eating IES-3 increased significantly. Finally, unlike existing diet-tracking apps, ours did not increase disordered eating; rather, for every disordered eating metric, the mean slightly decreased after the intervention, and there was a significant decrease in dietary restraint. However, due to the small sample size, the effects were small, and Holm-Bonferroni adjusted *P* values were not significant.

### Strengths

This work has several strengths. First, our study is the first intervention that we know of to provide a validated alternative to the expensive and time-consuming current approach of working one-on-one with a registered dietician, without the harm caused by most apps that emphasize weight loss and calorie counting. Second, our results demonstrated significant improvement in diet quality after a 4-week intervention using our app. Improved diet quality is associated with health promotion and disease prevention. Finally, our research used validated measures of intuitive eating, disordered eating, and psychological distress, as well as robustness checks, including bootstrapped CIs and nonparametric sensitivity analyses to strengthen confidence in the findings.

### Comparison With Prior Work

Prior work demonstrated the accuracy of logging meals via natural language within a mobile app [4] and showed that the use of intuitive eating principles may counteract disordered eating behaviors [13-16]. Our work builds upon this literature by investigating the use of a new mobile app that not only allows users to log meals with natural language but also promotes intuitive eating principles and asks users to log their hunger satiety.

### Limitations

Limitations of the study include the small sample size (n=45), which resulted in small effect sizes, and the lack of diversity because we only recruited college students, who have been shown in prior work to have inadequate nutrient intake [7,8]. Another limitation is the wide range of app use, resulting in study participants who did not use the app often despite the monetary incentive. Other limitations include the lack of a control group, the inclusion of participants with eating disorders and medically supervised diets, and the absence of socioeconomic data.

Future work on In2Eat will focus primarily on improving the app’s usability and engagement, as well as incorporating more intuitive eating features. On the basis of feedback from our study participants, we are currently adding computer vision and nutrition facts label scanning so users can more easily log their meals with photos of meals or nutrition facts labels. We are also adding tooltips with explanations for each nutrient and more useful feedback and visualizations, including a detailed analysis after logging a meal and a weekly diet quality score with feedback for improvement. Furthermore, we are expanding from iOS to the Android platform.

### Conclusions

To our knowledge, our In2Eat iOS app is the first mobile app to combine diet tracking with intuitive eating, and ours is the first study to demonstrate that the use of a diet-tracking app with a focus on intuitive eating instead of calorie counting improves diet quality without increasing disordered eating.

## Abbreviations

BCa: bias corrected and accelerated
CNN: convolutional neural network
DQQ: Diet Quality Questionnaire
EDE-Q: Eating Disorder Examination Questionnaire
IES-3: Intuitive Eating Scale version 3
K10: Kessler Psychological Distress Scale
LLM: large language model
NCD: noncommunicable disease
NCD-Protect: the degree to which dietary intake on the previous day protects against noncommunicable diseases
NCD-Risk: the degree to which dietary intake on the previous day increases risk for noncommunicable diseases
SUS: System Usability Scale
USDA: US Department of Agriculture

## Data Availability

The datasets generated and analyzed during this study are not publicly available due to institutional review board requirements regarding the privacy of sensitive personal data collected during this study, including mental health and food security.
